# Trends in CPAP adherence over twenty years of data collection: a flattened curve

**DOI:** 10.1186/s40463-016-0156-0

**Published:** 2016-08-19

**Authors:** Brian W. Rotenberg, Dorian Murariu, Kenny P. Pang

**Affiliations:** 1 Department of Otolaryngology – Head and Neck Surgery, Western University, London, ON Canada; 2 Asia Sleep Centre, Paragon, 290, Orchard Road, Unit 18-04, Singapore, 238859 Singapore

**Keywords:** Obstructive sleep apnea, CPAP, Adherence, Uvuloplataopharyngoplasty

## Abstract

**Background:**

Obstructive sleep apnea (OSA) is a common disorder, and continuous airway positive pressure (CPAP) is considered to be the gold standard of therapy. CPAP however is known to have problems with adherence, with many patients eventually abandoning the device. The purpose of this paper is to assess secular trends in CPAP adherence over the long term to see if there have been meaningful improvements in adherence in light of the multiple interventions proposed to do so.

**Methods:**

A comprehensive systematic literature review was conducted using the Medline-Ovid, Embase, and Pubmed databases, searching for data regarding CPAP adherence over a twenty year timeframe (1994–2015). Data was assessed for quality and then extracted. The main outcome measure was reported CPAP non-adherence. Secondary outcomes included changes in CPAP non-adherence when comparing short versus long-term, and changes in terms of behavioral counseling.

**Results:**

Eighty-two papers met study inclusion/exclusion criteria. The overall CPAP non-adherence rate based on a 7-h/night sleep time that was reported in studies conducted over the twenty year time frame was 34.1 %. There was no significant improvement over the time frame. Behavioral intervention improved adherence rates by ~1 h per night on average.

**Conclusions:**

The rate of CPAP adherence remains persistently low over twenty years worth of reported data. No clinically significant improvement in CPAP adherence was seen even in recent years despite efforts toward behavioral intervention and patient coaching. This low rate of adherence is problematic, and calls into question the concept of CPAP as gold-standard of therapy for OSA.

## Background

Obstructive sleep apnea (OSA) is a common disorder affecting 3–9 % of the general population and is well demonstrated to be a risk factor for resistant hypertension, fatal and non-fatal cardiovascular disease, neurological disease, and all-cause mortality [1]. OSA has predictable effects on decreasing economic outcomes, and is also a source of car accidents [1, 2]. Since the pervasive health effects of untreated OSA are so well described, practice parameters published by the American Academy of Sleep Medicine (AASM) recommend that continuous positive airway pressure (CPAP) should be considered both first-line and gold-standard treatment for OSA; many prominent published studies make similar statements [2–5].

When used as prescribed, CPAP reduces daytime sleepiness, normalizes sleep architecture, and improves numerous OSA-specific health outcomes [6]. The sense of CPAP being considered gold standard of therapy has become so entrenched within health that many North American remunerating agencies, be they government insurance or private plans, as well as in the medicolegal world, have adopted the position that patients must undergo a trial of CPAP prior to being considered for any other more invasive intervention. However, the AASM parameters also recognized that a significant proportion of patients are unable to tolerate CPAP therapy, and frequently seek alternate treatment [2].

Despite numerous advances in machine dynamics including quieter pumps, softer masks, and improved portability, adherence to CPAP continues to be a problem frequently encountered in clinician’s offices, with adherence rates generally ranging from 30 to 60 % [7, 8]. There are many reasons for this problem including comfort, convenience, claustrophobia, and cost. [2] It is also understood that many patients who start on a path to non-adherence frequently remain non-adherent and eventually abandon the machine altogether, with consequent return of symptoms and OSA-specific adverse consequences. Finally, although “optimal” adherence rates in the literature range from 4 to 6 h per night, it is becoming increasingly recognized that data used to define “optimal” or even “sufficient” use also very much related the outcome measure being studied as well as patient self-perception of their own OSA severity. Until recently such adherence data were underemphasized in the CPAP literature, giving the medical community at large a somewhat unrealistic sense of the effectiveness of the device as a blanket treatment for all comers with OSA. Perhaps consequently CPAP continues to stand at the top of the treatment modality spectrum for OSA despite the problematic shortcomings described above.

The purpose of the current study was to investigate whether or not there has been a change in CPAP adherence trends over the long term. This information is important when counseling patients about the likelihood of treatment success when a CPAP prescription is given.

## Methods

Our review was carried out in accordance with the preferred reporting items for systematic review and meta-analysis protocols (PRISMA-P) 2015 statement. A comprehensive systematic literature review was conducted using the Medline-Ovid, Embase, and Pubmed databases.

The primary search objective was to identify all papers reporting the results of clinical trials that used CPAP for the treatment of adults with OSA, and then to subsequently extract data on adherence if it was reported. The first step was a locate and review all of the studies listed for analysis in two major literature reviews, a Cochrane Collaboration review [9] and a second systematic literature review published by the National Institutes of Health Research (NIHR) [10] on the use of CPAP for the treatment of OSA. The second step was an extensive search of the PubMed/MedLine database, initiated using the following combined search terms (using both British and American spellings): “CPAP and obstructive sleep apnea” (*n* = 3058). From this list, studies were identified that (a) did not replicate studies already found (b) were otherwise eligible for inclusion and (c) comprise primary data, i.e. not reviews or guidelines. The third and final step was a review of all reference lists and tables of other studies found within papers identified in the second step. A PhD level biostatistician performed all three of the initial search steps. EBM rankings were used to assess data quality.

Articles were considered for inclusion into the study by reviewing the titles and abstracts of all retrieved studies. The senior study authors BWR and KPP did this and results were compiled to ensure no studies were missed. The full text of selected studies were then analyzed to ensure that the following inclusion criteria were met: diagnosis of OSA, no confounding data for central sleep apnea, and the paper referred to CPAP for treatment of OSA. Subsequently we reviewed the studies to ensure adherence data was reported, and that if reported it was by machine audit as opposed to patient-self-report.

## Results

**Fig. 1:**
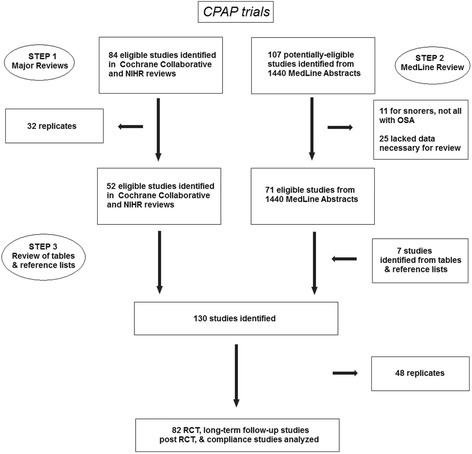
Prism chart showing article selection process

A total of 82 papers were identified for analysis. These included trials comparing CPAP versus sub-therapeutic (sham) CPAP [11–41], CPAP versus an oral placebo [40, 42–49], CPAP versus conservative or no therapy [17, 29, 50–60], CPAP versus an oral appliance [11, 12, 42, 57, 61–69], CPAP versus postural therapy [70–73], and CPAP alone assessing different means to modify adherence [15, 27, 37, 74–82]. The PRISMA chart summarizing the study flow is seen in Fig. 1.

### Overall CPAP adherence over the study duration

Sixty-six studies published between 1994 and 2015, inclusive, were identified in the literature that had CPAP adherence data reported as hours of use per night and were either a randomized clinical trial or a longitudinal cohort study specifically addressing the issue of CPAP adherence in adult patients with OSA. Sample sizes in these studies ranged from 13 to 356 (mean = 65, median = 37) subjects. The mean, non-weighted duration of nightly use was 4.6 h, from which the percentage of non-use, relative to optimum use, was calculated by subtracting 4.6 from 7.0 h, dividing this by 7.0 h and converting to a percentage; in this instance 34.1 % (i.e. the non-adherence rate). The weighted mean for nightly CPAP use was calculated by multiplying the mean use for each individual study by the number of CPAP users in that study, adding the resulting values from all 66 studies, and then dividing by the total number of subjects on CPAP across all studies; this yielded a weighted mean nightly CPAP use and percentage of use of 4.46 h/n and 36.3 %, respectively.

**Fig. 2:**
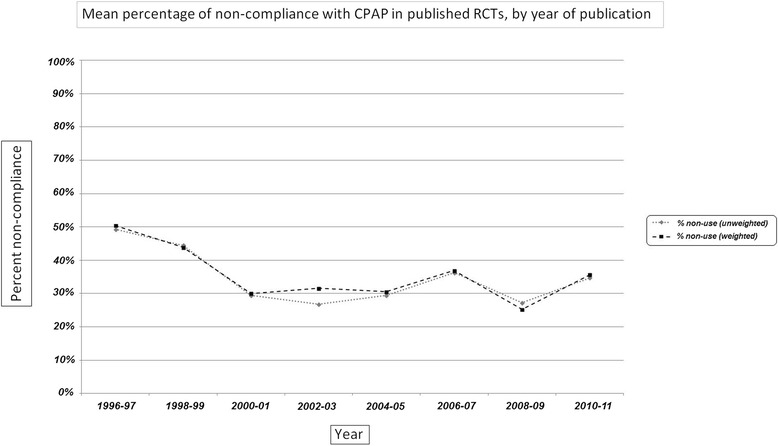
Graph showing percentage adherence over the yearsᅟ

Figure 2 depicts both the weighted and non-weighted mean percentage of non-use for all studies within each 2-year block starting with 1996–97. To incorporate studies from 2014, the final block, 2012–14, consists of 3 years. Note the initial decline in non-adherence from 1996 to 97 through 2000–01, after which there is no decline. In fact, non-adherence for 2012–14 was higher than for any other time period from 2000 to 01 onwards, possibly reflecting improved data reporting. Note also the almost identical curves for non-weighted and weighted data.

### Adherence when comparing short-term versus long-term

**Fig. 3:**
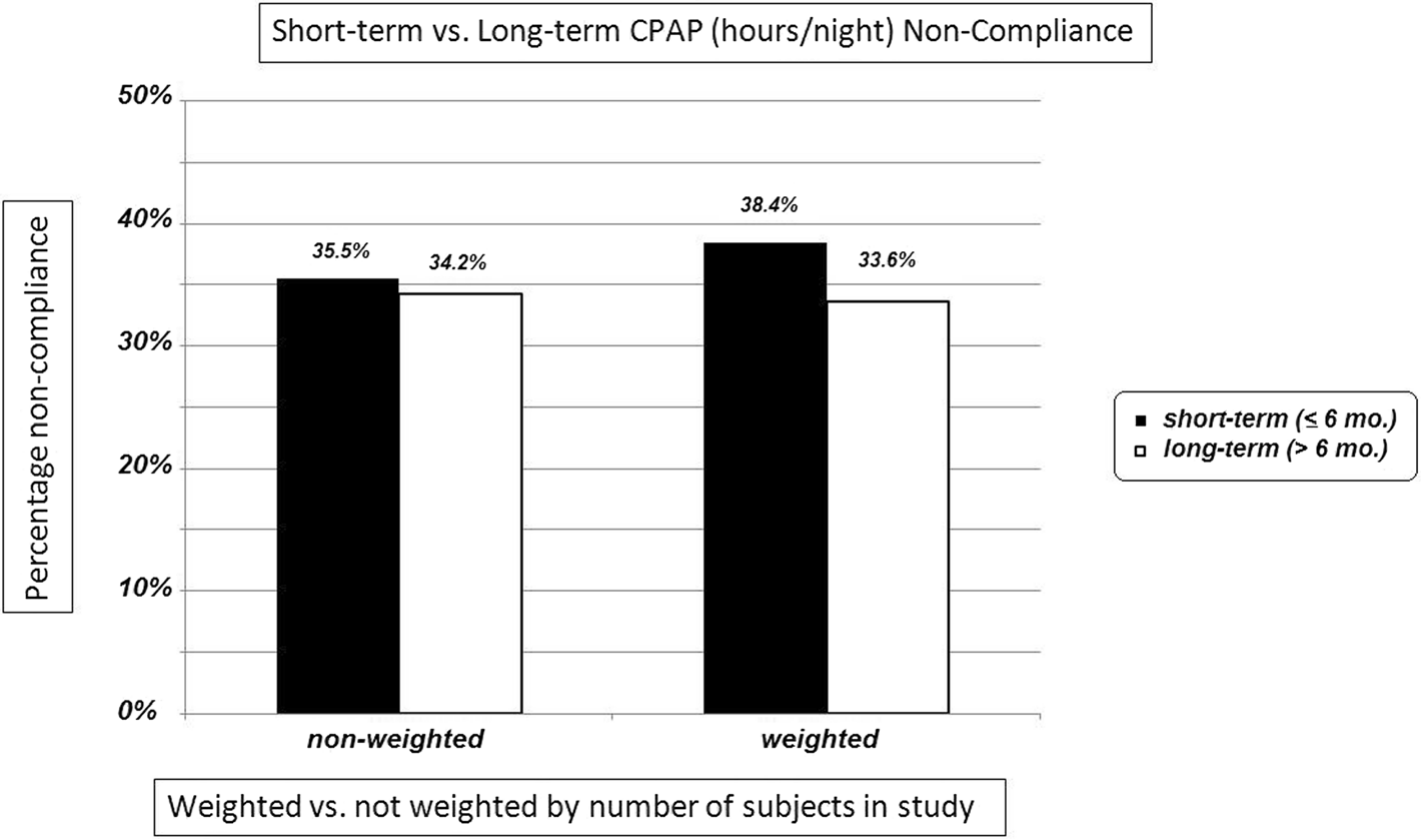
Bar chart showing percentage adherence cpmaring weight and non-weightedsubjects in the study

Eight studies were identified that had follow-up CPAP adherence assessments, including hours of CPAP use per night, 6 months or more since baseline, versus 64 studies with final assessments performed within the first 6 months. Again, both weighted and non-weighted means were calculated for mean nightly CPAP use and mean percentage of time CPAP was not used relative to the optimum 7.0 h per night. For the short-term studies, mean non-weighted and weighted mean values are 4.5 and 4.3 h/night, and 35.5 and 38.4 % CPAP non-use, respectively. Corresponding values for the long-term studies are 4.6 and 4.6, and 34.2 and 33.6 %, indicating slightly superior adherence at follow-up assessments performed in patients on CPAP for 6 months or more. This is reflected in Fig. 3.

### Adherence rates in self-report versus machine-interrogation

This analysis was restricted to studies in which measurements were made comparing both self-reported versus device-documented CPAP adherence values [79, 82]. In both studies, adherence was over-estimated by self-report, as summarized in Table 1. Note that for one of the studies, ‘percentage of adherence’ was the utilized measure of CPAP adherence, with adherence defined as four or more hours of use per night for 70 % or more of nights. For the other study, mean hours use per night was presented. The non-weighted percentage of self-reported over-estimation (relative to machine-documented use) ranged from roughly 25 to 50 %, averaging 35.4 %.

**Table 1:** Studies comparing self-reported vs. machine-documented CPAP compliance

1st author (year)	Outcome	Subjects (N)	Self-report	CPAP device	Absolute difference	Percent difference
Roecklein (2010) [79]	Hours per night at 3 months	28	4.68	2.35	2.33	49.8 %
Kribbs (1993) [83]	Hours per night over time	35	4.9	3.7	1.2	24.5 %
		311			Mean =	35.4 %
					Weighted mean =	32.7 %

### Adherence rates versus behavioral intervention

Several RCTs were identified in which some behavioural intervention was evaluated in terms of its ability to impact CPAP adherence, measured as hours of CPAP use per night [84–90], Although increased use was documented in three studies [84–86], four RCT did not find support for the behavioural intervention [87–90]. Table 2 summarizes these results.

**Table 2:** CPAP adherence versus behavioral intervention

1st author (year)	Study design	Subjects (N)	Intervention	Follow-up	CPAP use (h/n)		Significance
					Rx group	Controls	
Lo Bue (2014) [99]	RCT	20/20	“Reinforcing interventions”	1 year	4.3	3.8	NS
Lai (2014) [100]	RCT	50/50	Motivational enhancement	3 months	4.4	2.4	*p* < 0.001
Deng (2013) [101]	RCT	55/55	Stage-matched vs. standard care	3 months	5.65	5.26	*p* = 0.006
Olsen (2012) [102]	RCT	50/50	3 motivational interviews	3 months	4.63	3.16	*p* = 0.005
Roecklein (2010) [79]	RCT	13/15	Personalized feedback	3 months	2.35	1.97	NS
				Mean	4.3	3.3	28.6 % ↑
				Weighted mean	4.7	3.5	32.0 % ↑

## Discussion

CPAP is termed the gold standard for therapy of OSA and indeed when used as prescribed the health benefits are substantial. However this modality of treatment continues to be plagued by problems with adherence. The data identified in our review is the most up-to-date on the topic, and suggests that despite numerous interventions designed to improve adherence rates over the long term, secular trends do not show clinically impactful changes. The relatively poor adherence rates pre-2000 have not shown meaningful improvement over the ensuring 15 years, with overall rates stubbornly persistent at a high 30–40 % non-adherence.

The further problem identified by our data is that of the reporting of CPAP in clinical trials. In our review of 82 CPAP trials, 10.7 % of patients overall were unable to tolerate and thereby remain on CPAP over the duration of the trial in which they were participants, and the mean duration of nightly use was merely 4.7 h. This means that the average patient in bed for 7 h across these 83 closely supervised clinical trials (i.e. under the most optimal of circumstances and the best chance for success at the therapy) was not using it an average of 32.9 % of the time; extrapolating to 8-h nights, this time off CPAP rises to 41.3 %. When the nights per week of CPAP non-use have been examined, the percentages range from 10 to 40 % [12, 44, 56, 61, 70, 74–76], with one in three out of 25 patients in a very brief, 2-week cross-over trial by Ferguson et al. only using CPAP one out of every three nights or less [62]. These are highly alarming percentages, given that several published RCT have documented that at least a minimum level of CPAP use is required to reap benefits from it and that this therapeutic threshold generally falls between 5 and 6 h nightly [16, 43, 45, 74, 77]. It is, therefore, reasonable to assume that there is a sizeable subset, possibly a majority, of patients on CPAP who either cease to use it altogether, or fail to use it enough hours per night and/or nights per week to achieve clinically-significant benefits. This does call into question the validity of the results of some of these trials.

Prior to our study, Weaver et al. conducted the most recent prior review on CPAP as therapy for OSA in 2010, finding that there are many factors influencing CPAP adherence, both medical and non-medical (e.g. psychosocial), and that these need to be considered when both prescribing CPAP as well as designing effective interventions to improve adherence [7]. The authors wisely also pointed out that the cost-effectiveness of these interventions needs to be considered.

The most recent comprehensive literature review of CPAP adherence looks to be by Donovan et al. [91]. The authors investigated various outcomes including CPAP efficacy, behavioral interventions, and personalizing CPAP to patient need. The authors maintained that its main limitation is intolerability, which leads to low adherence. The authors call for more research on tailoring therapies to individual patients in order to enhance adherence.

The long-term effects of non-adherence bring to light the health related impact of untreated OSA. It is not sufficient to simply prescribe a CPAP machine and consider the patient to be treated. For example, BaHammam et al. [92] found that adherence to CPAP declined over a 10-month period, such that only 33 % of the OSA patients were considered to have “good adherence” by 10 months, even after receiving an educational intervention. Gagnadoux et al. [93] found that AHI scores and socioeconomic factors (employment and marital status) predicted mean CPAP adherence over several 6-month follow-up assessments. Martinez-Garcia et al. [94] follow a sample of older adults who were prescribed CPAP for up to 10 years and found that severe OSA not treated with CPAP was associated with a higher risk of cardiovascular death, and CPAP use decreased this risk (albeit non significantly). Stuck et al. [95] conducted a retrospective chart review of 750 patients who were prescribed CPAP and follow-up over a 2-year period to quantify several sleep-related events such as hours of sleep without CPAP, and the number of respiratory events with and without CPAP. These authors concluded that CPAP has a limited effectiveness, even among patients who are most adherent to treatment.

Although a detailed discussion of the effectiveness of interventions to improve CPAP adherence is beyond the scope of this current paper, several of the papers studied in our review did investigate these interventions [79, 92], including one review [96]. The strongest intervention, cognitive-behavioral, resulted in an increase of 1.44 h per night for participants in six studies. Both supportive and educational interventions were found to increase adherence to over 4 hours per night among study participants. Non-behavioral interventions for CPAP adherence, such as variants of CPAP, have also been investigated. A systematic review and meta-analysis found that patients preferred auto-CPAP over fixed pressure CPAP; however, there was no statistical difference in machine use [97]. Overall the authors did not find a difference in adherence between auto- and fixed-CPAP. Similarly, a more recent systematic review and meta-analysis found positive results associated with auto-CPAP over fixed-CPAP, including patient preference and enhanced adherence [98]. However, the latter did not found significant differences between the two variants in terms of AHI and ESS scores, leading the authors to question the clinical significance of their results.

As a systematic review, this study is limited to the quality of the included studies. Because it is a collection of findings from various other studies, it provides an overview of the direction of literature but is unable to show new findings. The authors recognize that in one study, CPAP was prescribed in patients with mild OSA, this might suggests that these patients who have no significant symptoms might not be compelled to use their CPAP device. It is also understandable that some patients may not sleep 7 h per night, as they are the working class group and may be too busy, and this might also affect the compliance percentage as the denominator is smaller. The older age group may have fragmented sleep patterns while the younger age group may not choose to wear their CPAP for social reasons. This study is also limited in the fact that only English language articles are considered, which may introduce a language bias. However, studies are published from a variety of centers internationally. Because this study is not a meta-analysis, study results have not been statistically combined for more powerful results. However, since adherence rates to CPAP are often measured differently, as are the defined outcome points for success, it is not feasible to directly compare papers in a statistically sound method.

## Conclusion

This review represents the most up to date data on secular trends in CPAP adherence. The findings are sobering. Our data suggest that despite numerous changes to machine and mask dynamics as well as behavioral interventions, CPAP adherence remains a severe problem for management of patients with OSA - the concept of CPAP as gold standard for OSA therapy is no longer valid. This paper’s data regarding a comprehensive assessment of CPAP adherence can be used when developing OSA treatment guidelines and when counseling patients about their OSA and the relative likelihood of treatment success for the various therapies at hand.
